# Impact of smoking on course and outcome of aneurysmal subarachnoid hemorrhage

**DOI:** 10.1007/s00701-020-04506-3

**Published:** 2020-07-30

**Authors:** H. Slettebø, T. Karic, A. Sorteberg

**Affiliations:** 1 Department of Neurosurgery, Oslo University Hospital, P.B. 0454 Nydalen, 0424 Oslo, Norway; 2 Department of Physical Medicine and Rehabilitation, Oslo University Hospital, P.B. 4950 Nydalen, 0424 Oslo, Norway; 3 Institute of Clinical Medicine, University of Oslo, P.B. 1072 Blindern, 0316 Oslo, Norway

**Keywords:** Intracranial aneurysm, Subarachnoid hemorrhage, Smoking, Outcome, Vasospasm, Complications

## Abstract

**Background:**

While the smoking-related risk of experiencing an aneurysmal subarachnoid hemorrhage (aSAH) is well established, it remains unclear whether smoking has an unexpected “protective effect” in aSAH, or if smokers are more at risk for complications and poor outcomes.

**Methods:**

Prospective, observational study investigating the course and outcome of aSAH in patients admitted during the years 2011 and 2012. Smoking status at admittance, demographic, medical, and radiological variables were registered along with management, complications, and outcome at 1 year in terms of mortality, modified Rankin score, and Glasgow outcome score extended. We compared current smokers with nonsmokers on group level and by paired analysis matched by aSAH severity, age, and severity of vasospasm.

**Results:**

We included 237 patients, thereof 138 current smokers (58.2%). Seventy-four smoker/nonsmoker pairs were matched. Smokers presented more often in poor clinical grade, had less subarachnoid blood, and were younger than nonsmokers. Ruptured aneurysms were larger, and multiple aneurysms more common in smokers. Severe multi-vessel vasospasm was less frequent in smokers, whereas all other complications occurred at similar rates. Mortality at 30 days was lower in smokers and functional outcome was similar in smokers and nonsmokers. Poor clinical grade, age, cerebral infarction, and vertebrobasilar aneurysms were independent predictors of 1-year mortality and of poor functional outcome. Serious comorbidity was a predictor of 1-year mortality. Smoking did not predict mortality or poor functional outcome.

**Conclusions:**

Notwithstanding clinically more severe aSAH, smokers developed less frequently severe vasospasm and had better outcome than expected. The risk for complications after aSAH is not increased in smokers.

## Introduction

Cigarette smoking is a well-documented risk factor for the formation, growth, multiplicity, and rupture of intracranial aneurysms: The risk of aneurysmal subarachnoid hemorrhage (aSAH) increases with the number of cigarettes smoked per day, and it has been estimated that 40% of aneurysmal ruptures can be attributed to smoking [20, 21, 34]. In a Norwegian study, only 19 of 107 aneurysmal subarachnoid hemorrhages occurred in never-smokers, while 82% of the patients were former or current smokers [50]. Smokers in general are estimated to be three to six times more at risk for aSAH than nonsmokers [20, 50]. Female smokers and heavy smokers may have an even higher risk, possibly 10 times as high as that in nonsmokers [30, 33]. There is evidence that cessation of smoking reduces the risk of aSAH [22].

While the smoking-related risk of experiencing an aSAH is well established, the influence of smoking status on the course and outcome of aSAH is less clear. Results from the relatively few reports are partly contradictory. Delayed neurological deterioration and “clinically confirmed vasospasm” have been reported to occur more often in smokers [20, 26, 28, 59].

Several authors have found similar or lower mortality in smokers than nonsmokers after aSAH [7, 14, 44, 51], and also functional outcome has been reported to be similar or superior in smokers [7, 14, 26, 62]. Smokers are younger when experiencing aSAH [7, 44, 59], a factor that could contribute to this paradoxal outcome in smokers.

It remains unclear whether smoking has an unexpected “protective effect” in aSAH, or if smokers are more at risk for complications and poor outcomes. To further elucidate this topic, we investigated a cohort of aSAH patients with regard to the course of disease and outcome in current smokers as compared with nonsmokers.

## Material and methods

### Patients

All patients admitted with aSAH to the Department of Neurosurgery, Oslo University Hospital, during 2011 and 2012 were included, and data were registered prospectively. Data not originally noted during enrollment were obtained from chart review or by phone, from patients or relatives to ensure correct smoking status.

Survivors were followed up for 1 year after their primary treatment, in order to register baseline characteristics, complications, and outcomes. Four years after the hemorrhage, the number of deaths was updated from the National Registry.

We used the following exclusion criteria: unknown smoking status, fusiform or mycotic aneurysms, unavailable or lost to follow-up (patients from outside our catchment area or those who refused follow-up), no written consent obtained. The data were acquired from a study approved by the Regional Committee for Medical Research Ethics 2011/2189 and a quality project approved by the institutional data protection officer (number 2011/20184).

### Institutional treatment principles

Our treatment algorithm has been previously described [54] and included pre-admission intravenous administration of tranexamic acid [15] and early aneurysm repair. We practiced extensive use of cerebrospinal fluid (CSF) drainage, in particular during the first week [9, 54]. We performed early tracheostomy in poor-grade sedated and intubated patients [49]. Control with computed tomography angiography (CTA) was performed routinely on day 7 after the ictus to detect vasospasm, respectively on day 5 in sedated and intubated patients. Transcranial Doppler (TCD) was also used to detect and follow the development of vasospasm [32]. We followed our standard treatment in mild to moderate vasospasm without clinical correlate, with cerebral perfusion pressure (CPP) maintained above 70 mmHg. In cases with severe vasospasm, the CPP was elevated to 90 mmHg by lowering resistance to CSF drainage, and if necessary, by adding hypertensive medication, and maintaining normovolemia. If clinical symptoms in severe vasospasm did not resolve, patients were treated with intraarterial nimodipine.

### Variables and statistics

“Smoker” was defined as a current smoker, meaning daily or occasional smoker, while the term “nonsmoker” was applied to a never-smoker or a former smoker.

The following data were registered: Demographic variables were specified, and medical comorbidity classified according to the Charlson Comorbidity Index [18]. Clinical status just prior to aneurysm repair or prior to intubation was expressed with the World Federation of Neurosurgical Societies (WFNS) grading system [48]. From the first available CT scan, we scored the amount of subarachnoid blood with the modified Fisher score [12], the presence and size of intracerebral hematomas (ICH), the amount of intraventricular blood (IVH) with a modified LeRoux score [29] where no IVH is represented by 0, and any midline shift. CTA provided aneurysm location, size, and multiplicity.

Treatment was classified as endovascular or surgical, and if both, registered as “surgical.” We registered if hemicraniectomy, tracheostomy, and pleural drainages were performed. We also registered time to aneurysm repair, time on invasive respiratory support, and length of stay (LOS).

Complications were noted: acute hydrocephalus (use of external CSF drainage and amount CSF drained), implantation of permanent CSF shunt, and pneumonia. Vasospasm was assessed with CTA in all patients and with TCD in selected patients and the highest grade of vasospasm found with either method was registered. Severe vasospasm was defined as > 50% narrowing of arterial diameter in more than one vessel on CTA [2] or digital subtraction angiography (DSA). Vasospasm on TCD was defined as moderate with a Lindegaard ratio between 3 and 6, and severe with a Lindegaard ratio > 6 [32].

New cerebral infarctions were diagnosed if new ischemic lesions were detected on CT or magnetic resonance (MRI) at discharge or at 3 months’ follow-up—be they secondary to hemorrhage, to aneurysm repair, to EVD insertion, or to vasospasm (regardless of cause).

Mortality was scored regardless of cause at 30 days, at 1 year, and in June 2016 (4 to 5 years after the ictus). Functional outcome was expressed at 1 year after the ictus with the modified Rankin scale (mRS) [3] and the Glasgow outcome scale extended [60].

### Statistics

Statistical analysis was performed in SPSS v 25.0 (IBM SPSS Statistics for Windows and Macintosh, version 25.0, Armonk, NY: IBM Corp). Normal distributed continuous variables are presented by mean and standard deviation; continuous variables which were not normal distributed are presented with median and range, whereas categorical variables are presented as frequencies or percentages. Smokers and nonsmokers were compared as independent groups using the independent samples *t* test (normal distribution), the Mann-Whitney *U* test (not normal distribution), or chi-square test (categorical data). We also matched pairs of smokers and nonsmokers by WFNS grade, age, and severity of vasospasm. We chose these variables because they are reported to be the strongest predictors of outcome after aSAH [14, 27]. Differences between matched pairs were calculated using the paired *t* test (normal distribution), Wilcoxon signed-rank test (not normal distribution), and McNemar test (categorical variables). Kaplan-Meier plots were used for survival and differences between subgroups were tested using the Mantel-Cox test. Univariate and multivariate regression analysis was performed to identify possible predictors of 1-year mortality and poor functional outcome. Any variable with *p* < 0.05 in the univariate analysis was included in the multivariable model, unless there occurred collinearity. A significance level of 5% was adopted and all *p* values are given for 2-sided tests.

## Results

### Patients and entry variables

**Fig. 1:**
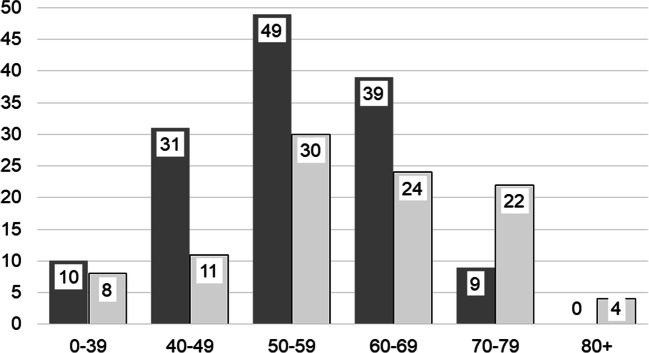
Age distribution (years) in smokers (dark bars) and nonsmokers (light bars). *n* = 237

During the study period, a total of 276 patients were admitted with aSAH. Thirty-nine of them were excluded due to insufficient information about smoking status, or because they would be unavailable for follow-up, or due to lack of informed consent. Hence, 237 patients were included in the study, 138 (58.2%) current smokers and 99 nonsmokers. Table 1 shows patient characteristics at admittance for smokers and nonsmokers. Smokers were on average 4 years younger than nonsmokers. Their age distribution is shown in Fig. 1.

**Table 1:** Patient characteristics; significant differences in italics

	Nonsmokers	Smokers	*p* value	Nonsmokers	Smokers	*p* value
Total number = 237	99	138 (58.2%)		74 matched case pairs		
Mean Age (years); (range) Median	59.4 (25–91) 60	55.0 (25–79) 56	0.004	55.9 (25–79) 56	55.9 (35–79) 56	0.979
Female	66 (66.7%)	85 (62.0%)	0.423	64.9%	64.9%	1.000
Mean body weight (kg); (range)	74.8 (42–125)	74.8 (43–149)	0.972	76.4 (42–125)	74.7 (45–125)	0.464
Comorbidity						
CCI (mean ± SD)	0.78 ± 1.33	0.57 ± 1.07	0.169	0.76 ± 1.33	0.57 ± 1.01	0.359
CCI ≥ 2	21.2%	14.5%	0.177	21.6%	16.2%	0.541
Hypertension	31.8%	31.1%	0.982	24.3%	29.6%	0.720
Anticoagulated at ictus	2.3%	7.9%	0.071	1.4%	6.8%	0.219
Use of platelet aggregation inhibitors at ictus	13.1%	10.1%	0.475	9.5%	12.2%	0.791
Rebleeding prior to aneurysm repair	13.5%	9.6%	0.344	13.9%	10.8%	0.804
WFNS grade						
1	39.4%	28.3%	0.072	39.2%		-------------
2	16.2%	21.7%	0.284	17.6%		
3	5.1%	2.9%	0.393	1.4%		
4	20.2%	15.9%	0.397	18.9%		
5	*19.2%*	*31.2%*	0.039	23.0%		

*CCI*, Charlson Comorbidity Index [18]; *WFNS*, World Federation of Neurosurgical Societies [48]

Smokers presented more often in WFNS grade 5 but had less subarachnoid blood than nonsmokers (Table 1). Smokers had larger ruptured aneurysms and presented more often with multiple aneurysms (Table 2). Multiplicity was more common in females (19.7% of females versus 6.1% males in nonsmokers and 27% females versus 14% males in smokers). This difference was not significant, but females had more than doubled risk for multiple aneurysms (OR 2.298, 95% CI 1.133–4.664, *p* = 0.021). Smokers had larger midline shifts on group level (although ICH was not different, Table 2). No other differences between smokers and nonsmokers were found for entry variables.

**Table 2:** Hemorrhage pattern and aneurysm characteristics; significant variables in italics

	Nonsmokers (*n* = 99)	Smokers (*n* = 138)	*p* value	74 matched case pairs		*p* value
				Nonsmokers	Smokers	
Modified Fisher [12] thick subarachnoid blood (3–4)	67.7%	62.3%	0.395	*66.2%*	*52.7%*	*0.042*
No intraventricular blood	27.3%	21.2%	0.263	25.7%	25.7%	1.000
LeRoux [29] ≥ 6	22.2%	26.3%	0.420	23.0%	24.3%	1.000
Intracerebral hemorrhage						
None	73.7%	67.4%	0.378	75.7%	64.9%	0.115
< 2 cm	9.1%	7.9%	0.760	8.1%	9.5%	1.000
2–5 cm	11.1%	13.8%	0.711	10.8%	18.9%	0.238
> 5 cm	6.1%	10.9%	0.199	5.4%	6.8%	1.000
Midline shift (mm; mean ± SD)	*0.87 ± 2.85*	*1.94 ± 4.59*	*0.042*	0.91 ± 2.69	1.54 ± 3.52	0.093
Aneurysm localization						
ACoA/ACA	39.4%	39.8%	0.943	39.2%	43.2%	0.701
MCA/ICA	41.4%	43.5%	0.751	39.2%	39.2%	1.000
Vertebrobasilar arteries	19.2%	16.7%	0.616	21.6%	17.2%	0.701
Aneurysm size (mm)						
Mean ± SD	*6.21 ± 3.86*	*7.95 ± 5.06*	*0.005*	*5.95 ± 3.91*	*8.24 ± 5.57*	*0.005*
Median (range)	*5.00 (1–20)*	*6.00 (2–29)*		*5.00 (1–20)*	*6.00 (2–29)*	
Multiple aneurysms	*15 (15.2%)*	*38 (27.5%)*	*0.024*	*11 (14.9%)*	*22 (29.7%)*	*0.043*
Number of aneurysms (mean ± SD)	*1.17 ± 0.43*	*1.43 ± 0.91*	*0.010*	*1.16 ± 0.41*	*1.50 ± 1.08*	*0.009*

*ACoA*, anterior communicating artery; *ACA*, anterior cerebral artery; *MCA*, middle cerebral artery; *ICA*, internal carotid artery

### Management and complications

Eighteen patients (ten smokers and eight nonsmokers) did not undergo aneurysm repair because they had cerebral circulatory arrest at arrival at our Department or because they were otherwise deemed unsalvageable; in one patient, aneurysm clipping was attempted but failed. All of them died within 10 days.

Among the 219 patients that underwent aneurysm repair, this was performed within 24 h after the ictus in 201 (91.8%). Aneurysm repair was deferred in patients with significant vasospasm on admission. Table 3 shows management variables and complications in smokers and nonsmokers for those that underwent aneurysm repair (*n* = 219). There were no significant differences in treatment variables and complications between smokers and nonsmokers apart from the frequency of severe vasospasm being lower in smokers (Table 3). Current smoking reduced the risk of severe vasospasm (OR 0.474 95% CI 0.237–0.947, *p* = 0.034), but was not a predictor of functional outcome or new cerebral infarction.

**Table 3:** Management and complications in 219 patients that underwent aneurysm repair; significant differences in italics

	Nonsmokers (*n* = 91)	Smokers (*n* = 128)	*p* value	74 matched case pairs		*p* value
				Nonsmokers	Smokers	
Time to repair (hours, median, range)	10.36 (0–283)	7.20 (0–312)	0.075	10.12 (0–283)	7.52 (0–118)	0.217
Surgical aneurysm repair	54%	47%	0.309	46%	47%	1.000
Time on invasive mechanical respiratory support (hours, median, range)	16.8 (2–1202)	27.1 (2–614)	0.350	8.26 (2–1202)	14.54 (1–614)	0.115
Length of stay (days; mean ± SD)	15.8 ± 10.4	15.2 ± 7.7	0.940	14.70 ± 10.51	14.08 ± 7.66	0.630
External ventricular drain	76%	79%	0.320	66%	70%	0.690
Cerebrospinal fluid drained (ml; mean ± SD)	2457 ± 1650	2364 ± 1579	0.720	2295 ± 1611	2402 ± 1695	0.971
Ventriculoperitoneal shunt < 1 year	27%	28%	0.785	23%	31%	0.359
Hemicraniectomy performed	4.4%	3.9%	0.863	5.8%	4.2%	1.000
Pleural drainage	12%	14%	0.661	12%	10%	0.804
Average number of pleural drains (if drained; mean ± SD)	1.55 ± 0.52	2.0 ± 1.80	0.330	1.67 ± 0.50	2.86 ± 2.73	0.543
Tracheostomy	33%	41%	0.205	30%	37%	0.359
Pneumonia	60%	63%	0.763	59%	56%	0.701
Vasospasm*						
None	41.9%	35.5%	0.349	38.0%		---------
Moderate in 1 vessel	12.8%	21.8%	0.096	16.9%		
Moderate in > 1 vessel	18.6%	28.2%	0.110	21.1%		
Severe in 1 vessel	10.5%	7.3%	0.414	11.3%		
Severe in > 1 vessel	*16.3%*	*7.3%*	*0.040*	12.7%		
New cerebral infarction	38%	40%	0.864	41.5%	40.3%	0.860

*Highest degree found on either cerebral computed angiography, digital subtraction angiography, or transcranial Doppler ultrasonography

### Outcome

**Fig. 2:**
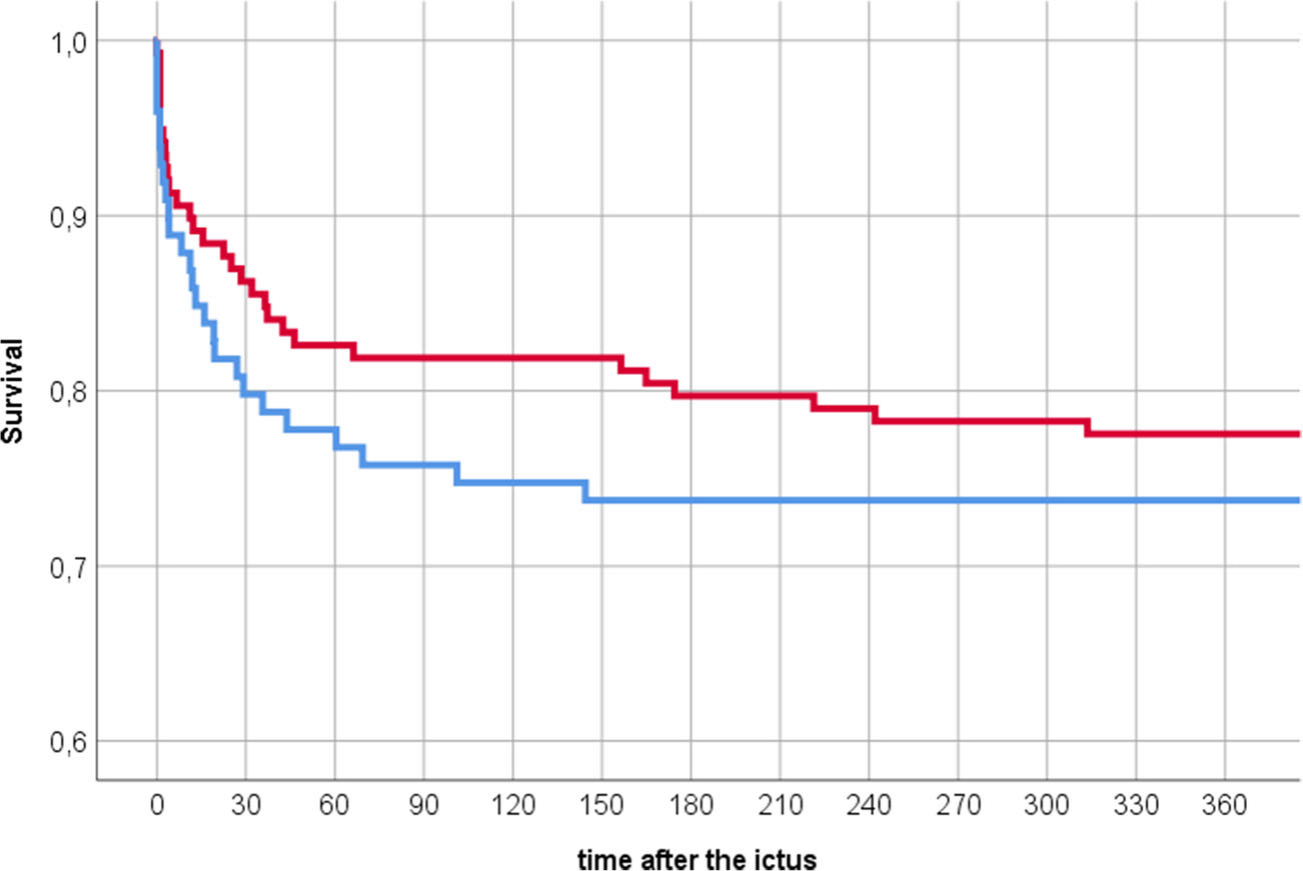
Kaplan-Meyer plot of 1-year survival in 237 patients with aneurysmal subarachnoid hemorrhage. Red line: smokers (*n* = 138); blue line: nonsmokers (*n* = 99); difference not statistically significant

Mortality regardless of cause in all 237 patients and in the 219 patients that underwent aneurysm repair was numerically higher in nonsmokers than smokers, but the difference was not statistically significant (Table 4). One-year survival for smokers and nonsmokers is illustrated in Fig. 2. When correcting for aSAH severity in the matched pairs analysis, mortality at 30 days was significantly lower in smokers (Table 4).

**Table 4:** Mortality in smokers and nonsmokers (%); significant differences in italics

	Nonsmokers	Smokers	*p* value
Mortality in all 237 patients			
At 30 days	20.2	13.8	0.188
At 1 year	27.3	22.5	0.500
At 1 year, WFNS 4 + 5 only	48.7	40.0	0.385
At last follow-up	30.3	26.8	0.556
Mortality in 219 patients with aneurysm repair			
At 30 days	13.2	7.0	0.099
At 1 year	19.8	16.4	0.164
At 1 year, WFNS 4 + 5 only	37.5	29.1	0.418
At last follow-up	24.2	21.1	0.590
Mortality in 74 matched case pairs			
At 30 days	*18.9*	*5.4*	*0.002*
At 1 year	24.3	13.5	0.096
At 1 year, WFNS 4 + 5 only	45.2	29.0	0.267
At last follow-up	28.4	17.6	0.115

*WFNS*, World Federation of Neurosurgical Societies [48]

Predictors of 1-year mortality in univariate analysis are shown in Table 5, left. Multivariate analysis identified WFNS grade 5 (OR 8.508, 95% CI 1.551–46.672, *p* = 0.014), cerebral infarction (OR 3.493, 95% CI 1.261–9.671, *p* = 0.016), age (OR 1.061, 95% CI 1.007–1.118, *p* = 0.025), ruptured aneurysm in the vertebrobasilar circulation (OR 4.463, 95% CI 1.288–15.467, *p* = 0.018), and serious comorbidity (CCI ≥ 2, OR 3.167, 95% CI 1.036–9.684, *p* = 0.043) as independent predictors of 1-year mortality. Smoking was not a risk factor of 1-year mortality; in fact, the OR was 0.813 albeit not being significant.

**Table 5:** Univariate analysis of predictors of 1-year mortality and of poor functional outcome (*n* = 237). Significant predictors are highlighted in italics

	Predictors of 1-year mortality		Predictors of poor functional outcome	
	Odds ratio (95% CI)	*p* value	Odds ratio (95% CI)	*p* value
Age at ictus	*1.079 (1.045–1.113)*	*0.000*	*1.094 (1.060–1.129)*	*0.000*
Sex (female)	1.384 (0.753–2.545)	0.296	1.234 (0.704–2162)	0.463
Current smoking	0.813 (0.446–1.482)	0.500	0.739 (0.427–1.278)	0.279
Body weight	1.010 (0.989–1.031)	0.312	0.999 (0.981–1.018)	0.927
Comorbidity CCI ≥ 2	*3.591 (1.768–7.294)*	*0.000*	1.947 (0.980–3.866)	0.057
Hypertension	1.539 (0.812–2.917)	0.186	1.680 (0.931–3.033)	0.085
Anticoagulated at ictus	1.623 (0.470–5.602)	0.444	2.069 (0.645–6.641)	0.222
Use of platelet aggregation inhibitors at ictus	1.383 (0.571–3.355)	0.473	2.014 (0.896–4.528)	0.090
Rebleeding prior to aneurysm repair	1.949 (0.813–4.671)	0.134	*3.305 (1.434–7.618)*	*0.005*
WFNS grade	*2.147 (1.686–2.734)*	*0.000*	*2.165 (1.748–2.682)*	*0.000*
Modified Fisher [12] thick subarachnoid blood (3–4)	*4.476 (2.004–9.999)*	*0.000*	*3.908 (1.990–7.673)*	*0.000*
LeRoux ≥ 6 [29]	*5.000 (2.599–9.619)*	*0.000*	*6.062 (3.199–11.488)*	*0.000*
Intracerebral hemorrhage	*1.493 (1.136–1.961)*	*0.004*	*1.852 (1.413–2.427)*	*0.000*
Midline shift	*1.136 (1.056–1.223)*	*0.001*	*1.251 (1.131–1.385)*	*0.000*
Aneurysm localization				
ACoA/ACA	Reference	-	Reference	-
MCA/ICA	*2.369 (1.116–5.026)*	*0.025*	1.846 (0.978–3.485)	0.059
Vertebrobasilar	*5.645 (2.393–13.313)*	*0.000*	*3.500 (1.600–7.657)*	*0.002*
Aneurysm size	*1.084 (1.021–1.152)*	*0.009*	*1.089 (1.027–1.154)*	*0.005*
Multiple aneurysms	1.034 (0.508–2.107)	0.926	0.956 (0.496–1.842)	0.892
Surgical aneurysm repair	0.572 (0.282–1.161)	0.122	1.083 (0.597–1.966)	0.794
Length of stay	*0.942 (0.905–0.981)*	*0.004*	0.984 (0.954–1.015)	0.302
External ventricular drain	*0.529 (0.285–0.982)*	*0.044*	0.895 (0.497–1.610)	0.710
ml cerebrospinal fluid drained	1.002 (0.996–1.009)	0.520	1.001 (0.996–1.007)	0.977
Ventriculoperitoneal shunt	0.994 (0.461–2.142)	0.916	*2.256 (1.173–4.339)*	*0.015*
Severe single or multi-vessel vasospasm	1.022 (0.412–2.537)	0.531	1.296 (0.616–2.727)	0.712
New cerebral infarction	*2.937 (1.338–6.445)*	*0.007*	*5.015 (2.565–9.806)*	*0.000*

*CI*, confidence interval, *CCI*, Charlson Comorbidity Index [18]; *WFNS*, World Federation of Neurosurgical Societies [48]; *ACoA*, anterior communicating artery; *ACA*, anterior cerebral artery; *MCA*, middle cerebral artery; *ICA*, internal carotid artery

**Fig. 3:**
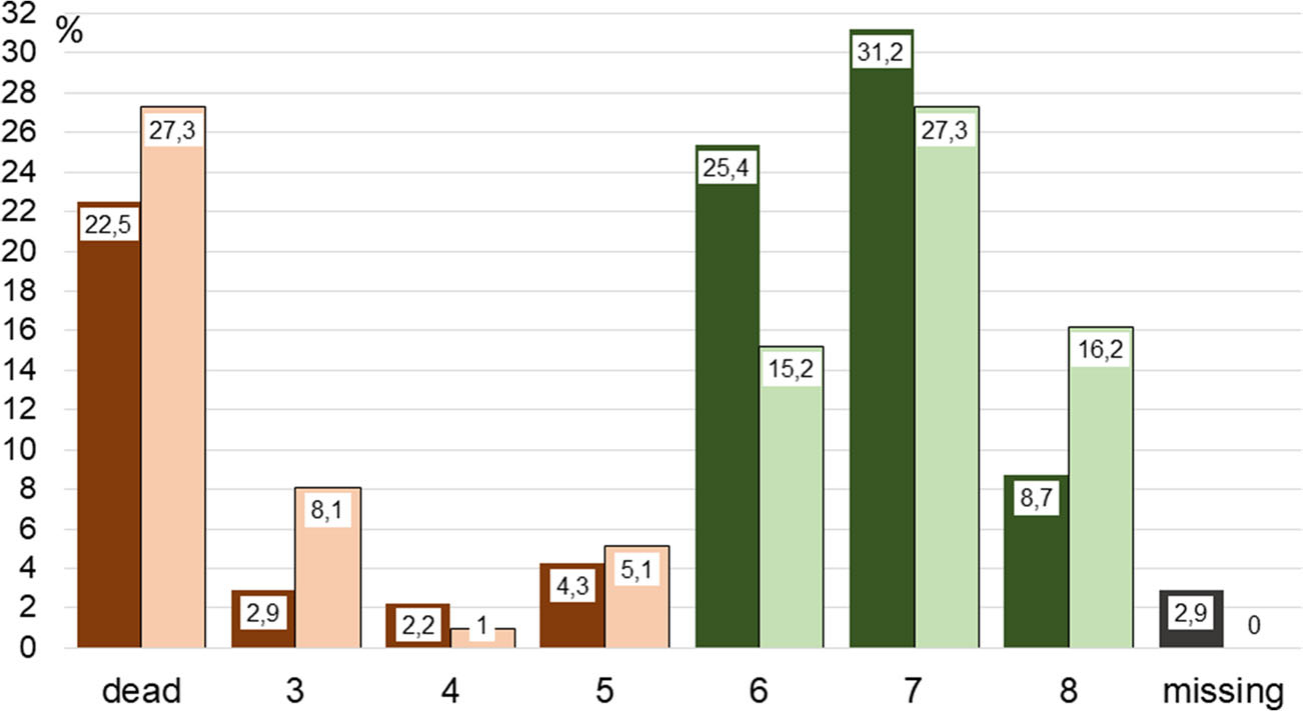
Functional status in terms of Glasgow outcome score extended [60] 1 year after the hemorrhage, *n* = 237. Dark bars: smokers; bright bars: nonsmokers. Brown bars indicate poor outcome; green bars indicate good outcome (lives independently)

**Fig. 4:**
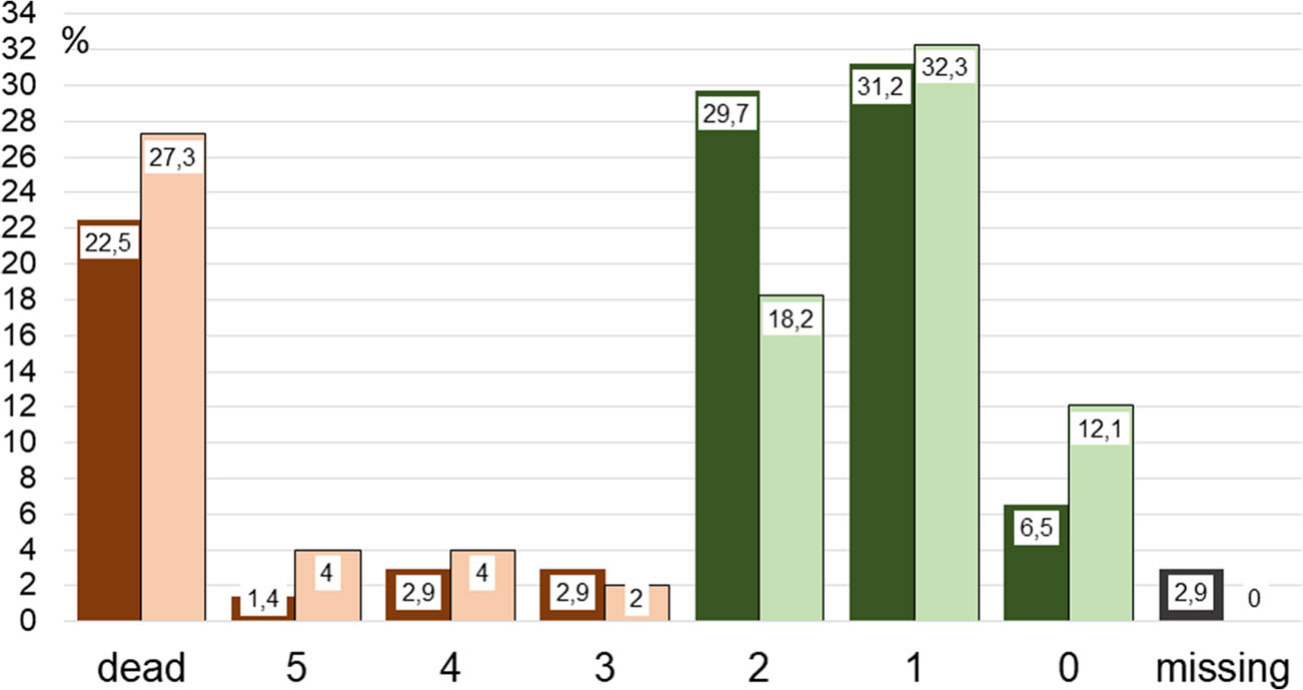
Functional status in terms of modified Rankin score [3] 1 year after the hemorrhage, *n* = 237. Dark bars: smokers; bright bars: nonsmokers. Brown bars indicate poor outcome; green bars indicate good outcome (lives independently)

Functional outcome at 1 year for all patients is presented as GOSE and mRS (Figs. 3 and 4). There was no significant difference in functional outcome between smokers and nonsmokers. Poor functional status (mRS 3–6) at 1-year was observed in 24.7% smokers and in 34.4% nonsmokers (23.6% in smokers and 33.8% in nonsmokers in the matched pairs analysis, respectively; *p* = 0.189). Predictors of poor functional status in univariate analysis at 1 year after the ictus are listed in Table 5, right. Multivariate analysis identified the following independent predictors: age (OR 1.103, 95% CI 1.052–1.156, *p* = 0.000); cerebral infarction (OR 4.723, 95% CI 1.922–11.606, *p* = 0.001); WFNS grade 5 (OR 8.264, 95% CI 2.104–32.461, *p* = 0.002) and grade 4 (OR 5.056, 95% CI 1.256–20.361, *p* = 0.023); midline shift (OR 1.189, 95% CI 1.022–1.384, *p* = 0.025); and ruptured aneurysm in the vertebrobasilar circulation (OR 3.285, 95% CI 1.051–10.264, *p* = 0.041).

## Discussion

The core findings of the present study was that smokers more often presented in WFNS grade 5, but had less subarachnoid blood than nonsmokers. Despite WFNS grades 4 and 5 multiplying the odds for mortality and poor functional outcome, smokers had lower 30-day mortality and their functional outcome was not inferior to the outcome in nonsmokers. Smokers were younger when their aneurysm ruptured, their ruptured aneurysms were larger, and they more often had multiple aneurysms.

### Patients and entry variables

Smokers presenting in a clinically poorer condition from aSAH than nonsmokers is a novel finding. The combination of higher WFNS grade and less subarachnoid blood in smokers is apparently self-contradictory. Yet, when an aneurysm ruptures, there is an instantaneous, immense increase in intracranial pressure (ICP) [41]. The extend and duration of this ICP increase is decisive for both the arrest of hemorrhage [41] and the level of consciousness at ictus. The risk of aSAH associated with smoking is greatest in the 3 h after nicotine intake [34], and considering the rise in blood pressure and pulse caused by smoking [13], this could translate into higher ICP levels upon aneurysm rupture in smokers. High ICP levels, in particular if maintained over a longer time period before declining, would cause coma and arrest subarachnoid bleeding more effectively. Such a scenario would also increase the number of sudden-death aSAH cases never reaching a hospital. In fact, Lindbohm et al. recently demonstrated that inclusion of sudden-death aSAH cases into mortality numbers reversed the paradox of higher survival in smokers [31]. In other words, more smokers die from their aSAH when considering all cases and findings of better survival in hospital-admitted cohorts seems to be biased.

Fifty-eight percent of our patients were current smokers, more than three times the prevalence of daily smokers in the general population, which was 17% in 2011—and twice the national prevalence reported 10 years earlier. Smokers were therefore clearly over-represented among our patients with aSAH. This is in agreement with findings in aSAH patients from North America and Europe, where 40–75% of patients have been smokers—compared with 20–35% in the general population [34, 58, 59]. Smoking rates have changed gradually in Norway during the last 50 years—from a maximum of three quarters of the male population smoking daily in the fifties, to about 12% of both genders today. Women in Norway have been fully emancipated as smokers for the last 20 years, corresponding to no difference in the fraction of females between our groups of smokers and nonsmokers. Given the association between smoking duration and multiple aneurysm formation [52], the Norwegian smoking emancipation may have contributed to our finding of multiple aneurysms being more common in smokers with a female preponderance. Schatlo et al. [52] found a clearly higher risk for multiplicity in males; however, their material included 44.3% patients with unruptured aneurysms. Other studies including only aSAH cases also found a link between multiple aneurysm formation and female sex [10, 19]. The incidence of aSAH is decreasing in the Nordic countries, possibly due to reduced smoking rates and due to improved treatment of high blood pressure [24, 30, 33].

Presently, smokers were younger than nonsmokers. The difference was though less than in earlier studies reporting smokers to be 5–10 years younger than nonsmokers at the onset of their hemorrhage [7, 44, 59]. Aneurysm location was very similar in smokers and nonsmokers, and overall, our material shows the same distribution as that found in recent series [11, 50], but ruptured aneurysm in the vertebrobasilar circulation was more common in our cohort than in that reported by Hammer at al. [14]

The ruptured aneurysms were presently larger in smokers than in nonsmokers. Two other studies reported similar findings [45, 61], whereas others did not find any difference in aneurysm diameter or rather increased odds for smaller aneurysms linked to smoking status [11, 16]. Given that many aneurysms show growth before eventually rupturing [53], and that smokers were younger when rupture occurred, one could speculate if there are biological factors in smokers leading to accelerated aneurysm growth. Such a mechanism could contribute to the increased risk of aneurysm rupture in smokers [50]. Inactivation of α-1-antitrypsin may also be a possible mechanism [56].

Hypertension is an established risk factor for aneurysm growth and for aSAH [2, 50]. We found hypertension to be equally frequent in our smokers and nonsmokers. Possibly similar factors leading to aneurysm growth could facilitate the formation of multiple aneurysms [10].

### Management and complications

Poor-grade aSAH was significantly more common in our smokers. Poor-grade patients are expected to have more complications and longer hospital stays [23] as well as a higher mortality, but we could not find significant differences in smokers and nonsmokers. Smoking, regardless of aSAH severity, is expected to imply a higher frequency of complications, since at least 10% of smokers have a smoking-attributable chronic disease, such as bronchitis, emphysema, or heart disease. A very large multi-center study found an OR of 1.45 for respiratory events after major surgery in current smokers [39].

Furthermore, the poorer admission status in smokers did not translate into more cerebral vasospasm or more new cerebral infarctions as compared with nonsmokers. On the contrary, severe vasospasm was found more frequently in nonsmokers than in smokers, whereas the frequency of new cerebral infarctions was similar. This may in part be caused by our inclusion of all new infarctions, also those that were procedure-related. The effect of smoking on the risk of cerebral infarction and vasospasm after aSAH is controversial; some have found an increased risk of cerebral infarction [37], symptomatic vasospasm [28, 59], or delayed clinical ischemia [8, 17, 26], while others found no impact of smoking on the risk of cerebral infarction or delayed cerebral ischemia [6]. Large amounts of SAH predispose for the development of vasospasm [12, 17], which could explain why nonsmokers presently had more frequently severe multi-vessel spasm, but one would have expected that also moderate vasospasm would be more frequent in nonsmokers, which was not the case. This finding also contradicts in part the notion that stiffer, more atherosclerotic blood vessel renders smokers less prone to vasospasm. Since poor clinical grade is a major predictor of DCI, we could have expected more ischemic events in our smoker group. de Rooij et al. [8] concluded in a meta-analysis that there was “strong evidence for an increased risk of DCI in smokers,” a finding that we could not corroborate. A possible explanation of this discrepancy is linked to the term DCI itself, since there was a large heterogeneity in what constitutes DCI in the studies analyzed by de Rooij et al. [8], in part using DCI interchangeably for clinical symptoms and radiological ischemia and/or radiological vasospasm. DCI and symptomatic vasospasm is often used interchangeably and it is the association between symptomatic vasospasm and smoking that mainly has been studied in the literature, whereas no clear relation has been established between smoking and the risk of radiological vasospasm [20]. Our study based vasospasm strictly on radiological and sonographic findings and the exclusive effect of smoking on severe multi-vessel spasm is novel. The lower frequency of this most dangerous form of vasospasm could have had an impact on our mortality numbers; however, there was a lower 30-day mortality in smokers in the paired analysis which was matched for vasospasm.

It may be possible that smoking, in addition to its harmful effects, has a beneficial effect in some acute vascular conditions. Several theories have been discussed. Smokers may have developed better collateral circulation or other compensatory mechanisms in response to chronic reduction in cerebral blood flow—mechanisms that may protect against secondary insults. Possible mechanisms for improved oxygen transport after acute smoking cessation are slightly higher hemoglobin values in smokers and the rapid reduction of hemoglobin-bound carbon monoxide [38, 40]. It has even been reported about a direct neuroprotective effect of nicotine through attenuation of oxidative stress and proinflammatory responses in Parkinson’s disease, spinal cord trauma, and neurotoxic insults [25, 47, 57]. Others have, however, shown that nicotine increases brain infarct size and worsens neurological deficits when ischemia is present [4]. The long-term effect of smoking cessation on aneurysm formation and rupture is not completely clarified. The results of Schatlo et al. [52] suggest that the risk of rupture is not reduced when quitting to smoke after the aneurysm has formed and that duration of smoking is important. The latter is in line with the findings of Can et al. [5], but they and Qureshi et al. [46] report a persistent increased risk of aneurysm rupture after smoking cessation. This contradicts Kim et al. [22] who report that aSAH is reduced to 59% after 5 years of smoking cessation. With such long-lasting impact of smoking on the natural history of intracranial aneurysms, one cannot exclude that also the protective effects persist over some time. This is partly supported by former smokers faring slightly better on some measures than never-smokers in the study of Dasenbrock et al. [7].

### Outcome in smokers

Serious comorbidity, age, and foremost clinical grade are strong predictors of mortality after aSAH [27, 51]. We also know that life expectancy in smokers is in general 10 years shorter than in never-smokers, premature deaths being caused by cardiovascular disease, cancer, or respiratory disease [58]. We used the Charlson Comorbidity Index to describe comorbidity, which was similar in smokers and nonsmokers. Only 17% of all our patients had a high CCI (≥ 2) and there was no difference between smokers and nonsmokers. A CCI ≥ 2 or more has been shown to predict a relatively high risk of death within 6 months in stroke patients [18], which concurs with our findings at 1 year. With poor-grade patients more often being smokers in the present study, one would anticipate mortality to be higher than in nonsmokers. Even though not being significant, lower mortality numbers were found 1 year after the ictus in smokers. This unexpected finding is in line with the study of Zheng et al. [62] who found a borderline significant higher chance of good outcome in smokers in poor-grade aSAH (WFNS 4 and 5). The effect of worse clinical grades among smokers may have been outweighed by the positive effects of their younger age. One could, however, debate if our age difference of median 55 years in smokers versus 59 years in nonsmokers is clinically relevant. Moreover, in the paired analysis matched for age, the lower mortality in smokers persisted and even became significantly lower at 30 days. Several authors have found better outcomes in smokers compared with nonsmokers in different settings, including aSAH [1, 7, 14, 35, 44] an effect that could not be explained by younger age alone. “Paradoxically superior outcomes” in smokers have also been reported after myocardial infarction and after acute ischemic stroke, but may be due to residual confounding or lower baseline risks [1, 35]. Nevertheless, it seems that worse clinical grades in smokers had less impact on outcomes than in nonsmokers, since the outcomes in the two groups were similar. A contributing factor could be that the rate of rebleed presently was somewhat lower in smokers since a rebleed can triple 1-year mortality after aSAH [42, 55]. On the other hand, rebleed was not an independent predictor of 1-year mortality in our study.

Cerebral vasospasm is one of the most feared complications to aSAH, potentially leading to poor outcome. The lower frequency of severe vasospasm in the present study may have contributed to similar functional outcomes in smokers and nonsmokers, compensating for worse clinical grade in the smokers. Grade of vasospasm or severe vasospasm was though not a predictor of mortality or functional outcome in the present study and poor functional outcome was numerically less frequent in smokers in the paired analysis matched for WFNS grade, age, and vasospasm. Hammer et al. [14] found smoking (OR: 0.21; *p* = 0.0031) and hypertension (OR: 0.18; *p* = 0.0019) to reduce the risk of poor clinical outcome of aSAH after 1 year. Smoking was not a predictor of poor functional outcome in the present study.

Overall, smokers and nonsmokers have similar outcomes after aSAH, which is in agreement with recent findings and is also corroborated by the similar comorbidities, complications, and hemorrhage pattern in our two study groups [6, 27, 36, 43]. Still, these results need to be interpreted in light of patient selection, merely comprising hospital-admitted aSAH cases.

### Limitations and strengths

We registered merely current smoking status and not if nonsmokers have ever smoked and how long time it was since they abandoned smoking. There may be differences in former smokers and never-smokers. We also did not control for any nicotine replacement therapy during hospitalization. However, nicotine replacement therapy was very uncommon at our department during the study period so that any effect probably is negligible. A strength was that data were acquired from an observational prospective study, with robust follow-up data and little drop-out/missing data. We also have a well-established institutional treatment protocol ensuring standardized treatment of all patients and thereby similar treatment of both study groups. By including 31 variables, our study combines a larger amount of neurosurgical and medical variables than other studies and may thereby contribute with a more comprehensive approach. Furthermore, more detailed analysis of some variables such as radiological vasospasm disclosed a more nuanced pattern, thereby partly disentangling apparently conflicting literature on the matter.

## Conclusions

Notwithstanding clinically more severe aSAH, smokers developed less frequently severe multi-vessel spasm and had better outcome than expected. The risk for complications after aSAH is not increased in smokers.

## Abbreviations

ACA: Anterior cerebral artery
ACoA: Anterior communicating artery
aSAH: Aneurysmal subarachnoid hemorrhage
CT: Computed tomography
CTA: Computed tomography angiography
CPP: Cerebral perfusion pressure
CSF: Cerebrospinal fluid
DCI: Delayed cerebral ischemia
GOSE: Glasgow outcome score extended
ICA: Internal carotid artery
ICH: Intracerebral hemorrhage
IVH: Intraventricular hemorrhage
ICP: Intracranial pressure
MCA: Middle cerebral artery
MRI: Magnetic resonance imaging
mRS: Modified Rankin score
TCD: Transcranial Doppler ultrasonography
WFNS: World Federation of Neurosurgical societies
